# Tannic Acid-Decorated Bimetallic Copper–Gold Nanoparticles with High Catalytic Activity for the Degradation of 4-Nitrophenol and Rhodamine B

**DOI:** 10.1021/acsomega.4c02036

**Published:** 2024-06-03

**Authors:** Cheng-Chih Liu, Wei-Yu Wang, Cho-Chun Hu, Tai-Chia Chiu

**Affiliations:** Department of Applied Science, National Taitung University, 369, Section 2, University Road, Taitung 950309, Taiwan

## Abstract

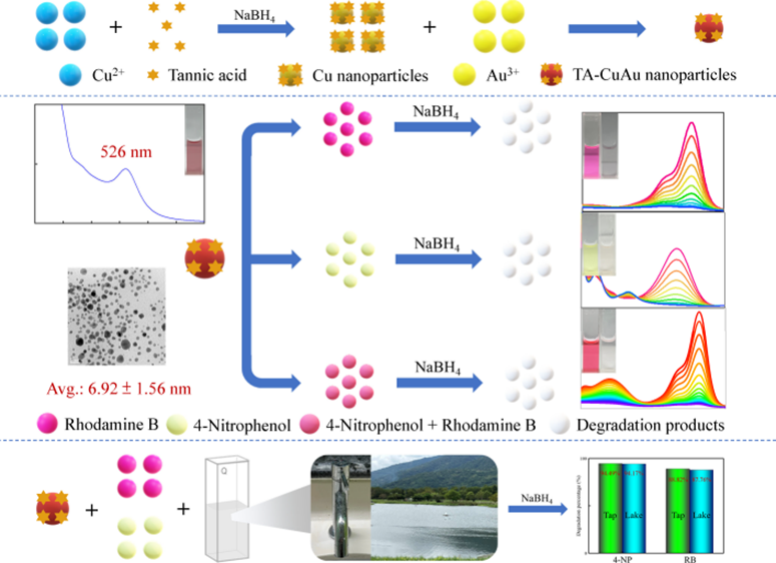

In this study, tannic acid (TA) was applied as a stabilizing agent for synthesizing bimetallic copper–gold (CuAu) nanoparticles. Cu(NO_3_)_2_ and NaAuCl_4_ were used as the sources of copper and gold ions, respectively, and NaBH_4_ was employed as a reducing agent. The prepared TA-CuAu nanoparticles were extensively characterized via ultraviolet–visible spectroscopy, Fourier transform infrared spectroscopy, transmission electron microscopy, X-ray diffraction, and zeta potential analyses. To evaluate their catalytic activity, the TA-CuAu nanoparticles and NaBH_4_ were applied in the degradation of 4-nitrophenol (4-NP) and rhodamine B (RB) individually and in a mixture. The individual degradation of 4-NP and RB was completed within 10 min, and the apparent rate constants were calculated as 0.3046 and 0.2628 min^–1^, respectively, emphasizing the efficient catalytic activity of the TA-CuAu nanoparticles. Additionally, controlled experiments were performed for the degradation of 4-NP and RB in the absence of catalysts or NaBH_4_ to investigate the kinetic feasibility of the catalytic reactions. A mixture of 4-NP and RB was successfully degraded within 10 min using the TA-CuAu nanoparticles as catalysts. Furthermore, the reuse of the catalysts after five successive cycles demonstrates an outstanding performance with no significant loss in the catalytic activity. Finally, the successful treatment of the tap and lake water samples spiked with 4-NP and RB using the TA-CuAu nanoparticles further confirmed their application potential as catalysts in environmental water remediation.

## 1 Introduction

Over the past few decades, owing to the rapid development of modern industry and technology, environmental pollution has emerged as a major issue for human wellness. Therefore, protection of the global environment is gaining considerable importance because of the continuous production of hazardous and toxic pollutants. Among various pollutants, organic dyes and nitroaromatic compounds are extensively utilized as intermediates in pharmaceutical and textile industries for preparing drugs, dyes, pigments, plastics, pesticides, and explosives.^1,2^ These toxic chemicals are often highly stable, nonbiodegradable, and carcinogenic, causing adverse effects on the environment and human health upon exposure. Various strategies have been developed for their degradation, such as electrolytic oxidation,^3^ photocatalytic degradation,^4^ and catalytic hydrogenation.^5^ Therefore, the synthesis of high-activity catalysts is essential for the efficient degradation of environmental pollutants to low- or nontoxic products that can be widely applied as precursors to manufacture various chemicals.

Over the past few decades, owing to the high surface-to-volume ratio, large surface area, and unique electronic properties, metal nanoparticle-based catalysts have exhibited excellent catalytic activity in hydrogenation and oxidation.^6−8^ In addition to their surface properties, these catalysts exhibit significant capability for sensing various analytes.^9−11^ These nanoparticles are easy to prepare and are cost-effective. Furthermore, they exhibit tunable catalytic activity through control of the particle size for degrading organic pollutants. However, bare metal nanoparticles tend to rapidly aggregate because of their high surface energy, losing their catalytic activities.^12^ To prevent the nanoparticle aggregation, various types of capping agents, such as polyvinylpyrrolidone,^13^ asparagine,^14^ gallic acid,^15^ lignin,^16^ and sodium dodecyl sulfate,^17^ have been applied to functionalize and stabilize the metal nanoparticles. These capping molecules stabilize the metal nanoparticles, extending their life span. Additionally, they manipulate the surface charge, surface area, and redox potential of the metal nanoparticles. However, they reduce the interfacial contact between the target analytes and metal nanoparticles and induce a diffusion barrier, which reduces the catalytic activity of the nanoparticles. Therefore, the selection of suitable capping molecules is important for improving the stability and catalytic activity of metal nanoparticles.

Compared with bulk materials, noble metal (Au, Ag, Pt, and Pd) nanoparticles exhibit distinct catalytic activities in the presence of NaBH_4_, owing to their high surface-to-volume ratios and large surface areas.^18,19^ Additionally, bimetallic nanoparticles show considerable advantages in terms of physical and chemical properties, such as high stability, easy functionality owing to cooperative and synergistic effects, and high activity, compared with their individual metallic nanoparticles.^19^ Bimetallic nanoparticles can be synthesized as either alloys or core–shell structures, depending on the simultaneous or consecutive reduction of metal ions, respectively. However, the high cost of these metal salts significantly limits their practical applications. Copper is an earth-abundant and inexpensive metal, and it has been used as a competitive alternative to noble metals.^20^ The combination of copper with noble metals for synthesizing bimetallic nanoparticles has gained significant attention to enhance the catalytic activity of component materials. Yin et al.^21^ synthesized nanoalloyed gold–copper (AuCu) nanoparticles under controlled thermochemical conditions and applied them as bifunctional catalysts for CO oxidation and oxygen activation. Hoffman et al.^22^ reported the synthesis of bimetallic AuCu nanoparticles with a tunable composition and an average size of 2.0 nm. The ultrasmall nanoparticles were transferred from organic to aqueous solutions by complexing dodecanethiol with sodium dodecyl sulfate. They exhibit potential for application as catalysts in CO_2_ reduction. Boeva et al.^23^ prepared bimetallic AuCu nanoparticles that exhibited higher catalytic activity for the H_2_/D_2_ exchange reaction than monometallic Au and Cu nanoparticles. The AuCu nanoparticles also demonstrated excellent stability. Ahmad et al.^24^ prepared bimetallic Cu–Ag nanoparticles decorated on poly(cyclotriphosphazene-*co*-4,4′-sulfonyldiphenol) nanotubes, which exhibited higher catalytic activity for the catalytic reduction of 4-nitrophenol (4-NP) than their constituent monometallic nanoparticles. Besides, to further improve the catalytic performance, metal nanoparticles deposited on the support have been developed.^25,26^ Two-dimensional membrane materials, serving as carriers for noble metal nanoparticles, offer numerous advantages such as enhanced catalytic performance and catalyst stability.

**Scheme 1:**
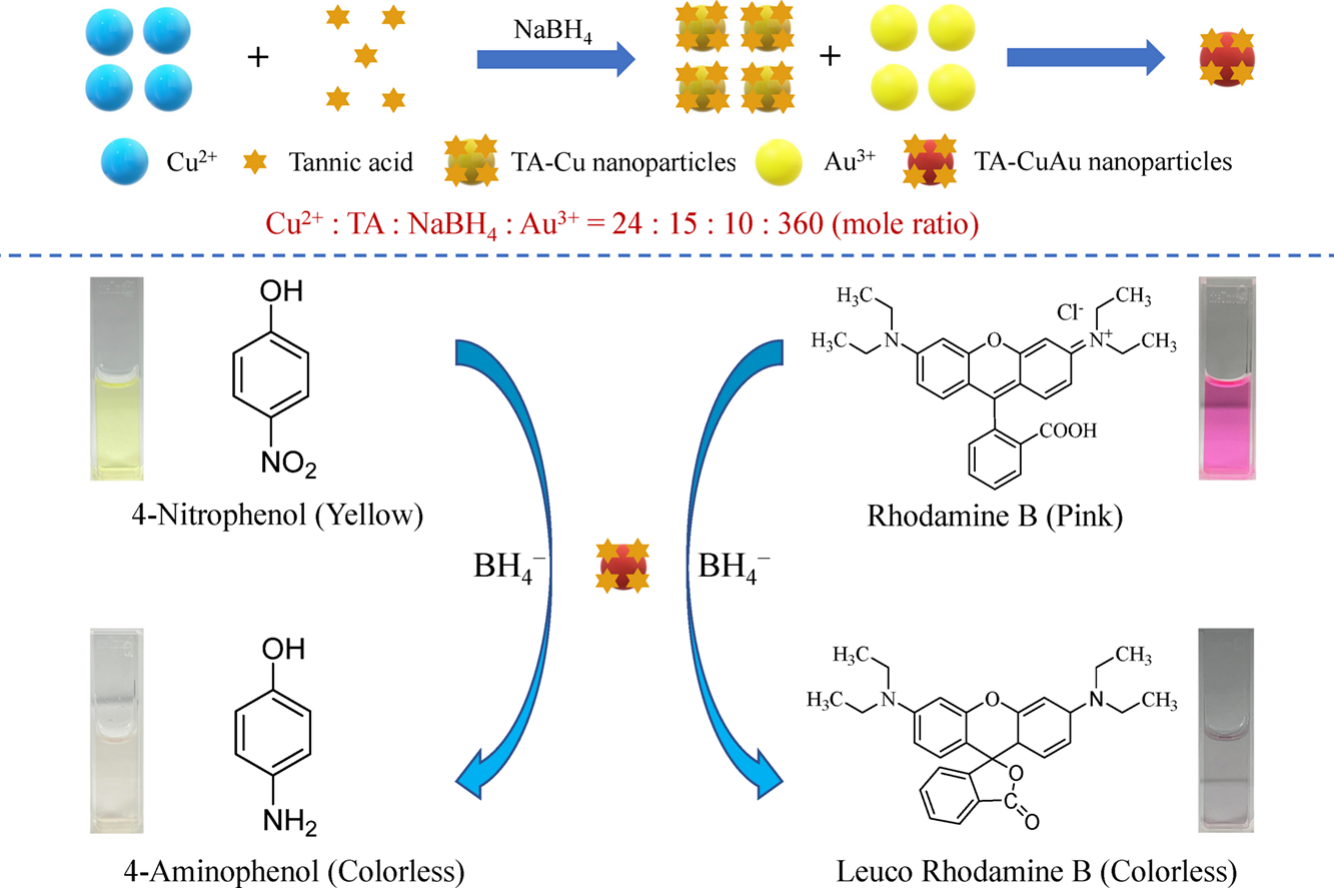
Schematics of the Preparation of the TA-CuAu Nanoparticles and Their Application as Catalysts for the Degradation of 4-NP and RB

Tannic acid (TA) is a biocompatible plant-derived polyphenolic biomolecule. TA can be used as an effective reducing and capping agent in the synthesis of metal nanoparticles and can functionalize the nanoparticle surfaces through covalent and noncovalent interactions.^27−29^ This study demonstrated a facile and efficient strategy for preparing CuAu nanoparticles using TA as a stabilizer and NaBH_4_ as a reductant. The resulting TA-stabilized CuAu (TA-CuAu) nanoparticles were applied as catalysts for the degradation of 4-NP and rhodamine B (RB) (Scheme 1). The structural and morphological characteristics, such as shape, size, functional groups, and surface charge, of the prepared CuAu nanoparticles were investigated through ultraviolet–visible (UV–vis) spectroscopy, Fourier transform infrared (FTIR) spectroscopy, transmission electron microscopy (TEM), X-ray diffraction (XRD), and zeta potential analyses. The catalytic activity of the bimetallic CuAu nanoparticles was evaluated in terms of their degradation potential toward 4-NP and RB in the presence of NaBH_4_. Several experimental parameters, such as the time-based stability and dependence of degradation capability on the catalytic dosage and NaBH_4_ amount, were also investigated.

## 2 Experimental Section

### 2.1 Chemicals and Materials

All reagents used in this study were of analytical grade and were used as received without further purification. TA was purchased from Acros Organics (Geel, Belgium). NaAuCl_4_·2H_2_O, Cu(NO_3_)_2_·3H_2_O, and NaBH_4_ were obtained from Sigma-Aldrich (St. Louis, MO, USA). HCl was obtained from Aencore (Surrey Hills, Australia). 4-NP and 4-aminophenol were purchased from Alfa Aesar (Ward Hill, MA, USA). Tris(hydroxymethyl) aminomethane (Tris) was acquired from J.T. Baker (Phillipsburg, NJ, USA). RB was purchased from Tokyo Chemical Industry (Tokyo, Japan). Tris (0.6067 g) was dissolved in 50 mL of deionized (DI) water, and the pH of the solution was adjusted from 5.0 to 9.0 with 0.1 M HCl to prepare a 100 mM Tris–HCl buffer with pH 5.0–9.0.

### 2.2 Preparation of TA-Capped CuAu Nanoparticles

The TA-CuAu nanoparticles were synthesized using a previously reported method with slight modifications.^30^ In a typical experiment, Cu(NO_3_)_2_ (1 mL; 1 mM) was added to TA (2 mL; 0.5, 1.0, 1.25, 2.5, and 5.0 mM) aqueous solutions. Afterward, a freshly prepared and ice-cooled NaBH_4_ solution (6 mL; 0.5, 1.0, 2.5, 5.0, and 8.0 mM) was added to the system under rapid stirring. Consequently, the solution turned pale yellow. After approximately 20 min, an aqueous HAuCl_4_ solution (1 mL; 1, 5, 10, 15, and 18 mM) was added, and the solution turned wine red. The solution was continuously stirred for another 40 min. The final solution was stored in a refrigerator at 4 °C until further applications.

### 2.3 Sample Characterization

UV–vis spectra were recorded in the wavelength range of 200–600 nm using an Analytik Jena Specord 210 Plus (Analytik Jena, Jena, Germany) spectrophotometer. FTIR spectra were obtained in the transmission mode using a Nicolet iS5 spectrometer (Thermo Fisher Scientific, Waltham, MA, USA) using KBr pellets. TEM images were acquired by using a JEM-3010 TEM instrument (JEOL, Tokyo, Japan). XRD patterns were measured by using an X-ray diffractometer (D2-Phaser, Bruker, Karlsruhe, Germany). X-ray photoelectron spectroscopy (XPS) analysis was performed on an XPS system (Thermo Fisher Scientific, Waltham, MA, USA) with a monochromatic Al Kα X-ray source (1486.6 eV) to reveal the surface elements and chemical states of the catalysts. Dynamic light scattering and zeta potentials were determined using a Zetasizer Nano ZS90 particle size analyzer (Malvern Panalytical, Malvern, U.K.).

### 2.4 Catalytic Degradation of 4-Nitrophenol and Rhodamine B

The catalytic activity of the TA-CuAu nanoparticles for the degradation of 4-NP and RB was explored in the presence of NaBH_4_. The reactions were evaluated in a 4 mL quartz cuvette with a 1.0 cm optical path length. Briefly, for 4-NP degradation, a solution of DI water (1400 μL), 4-NP (1 mM; 200 μL), and TA-CuAu nanoparticles (50 μg/mL; 200 μL) was mixed with a freshly prepared NaBH_4_ solution (10 mM; 200 μL) in a 4 mL quartz cuvette. For RB degradation, a solution of DI water (1400 μL), RB (0.1 mM; 200 μL), and the TA-CuAu nanoparticles (50 μg/mL; 200 μL) was mixed with a freshly prepared NaBH_4_ solution (3 mM; 200 μL) in a 4 mL quartz cuvette. For analyzing the progression of catalytic reactions, the absorption spectra of the reaction solutions were recorded within the spectral ranges 200–500 nm for 4-NP and 400–600 nm for RB, with a scan interval of 1.0 min. The degradation efficiency and rate constant for the catalytic process were determined by measuring the change in absorbance at 400 and 554 nm as a function of the reaction time for 4-NP and RB, respectively. The simultaneous degradation of 4-NP (0.25 mM; 200 μL) and RB (0.1 mM; 400 μL) in their mixture was also investigated to determine the catalytic performance of the TA-CuAu nanoparticles in multiple-pollutant degradation.

## 3 Results and Discussion

### 3.1 Optimization of Synthesis Conditions

A chemical reduction approach was used for the preparation of the CuAu nanoparticles, and copper nitrate and sodium tetrachloroauric salt were used as the precursors of copper and gold ions, respectively, as shown in Scheme 1. NaBH_4_ acted as a reductant in the reduction of Cu^2+^ ions into Cu atoms and the subsequent reduction of Au^3+^ to Au atoms that were coagulated to form the CuAu nanoparticles. TA was used as a stabilizing agent to prevent the aggregation of the metal nanoparticles.^27,28^ Different variables affecting the synthesis of nanoparticles, such as the concentrations of Au^3+^, TA, and NaBH_4_, were optimized to achieve high catalytic activities of the TA-CuAu nanoparticles. UV–vis absorption spectroscopy was employed to investigate the formation and catalytic activity of the TA-CuAu nanoparticles. Notably, the degradation performance of the TA-CuAu nanoparticles improved after optimizing the concentrations of Au^3+^, TA, and NaBH_4_ to 15 (Figure S1), 1.25 (Figure S2), and 5 mM (Figure S3), respectively.

### 3.2 Characterization of the TA-Capped CuAu Nanoparticles

**Figure 1:**
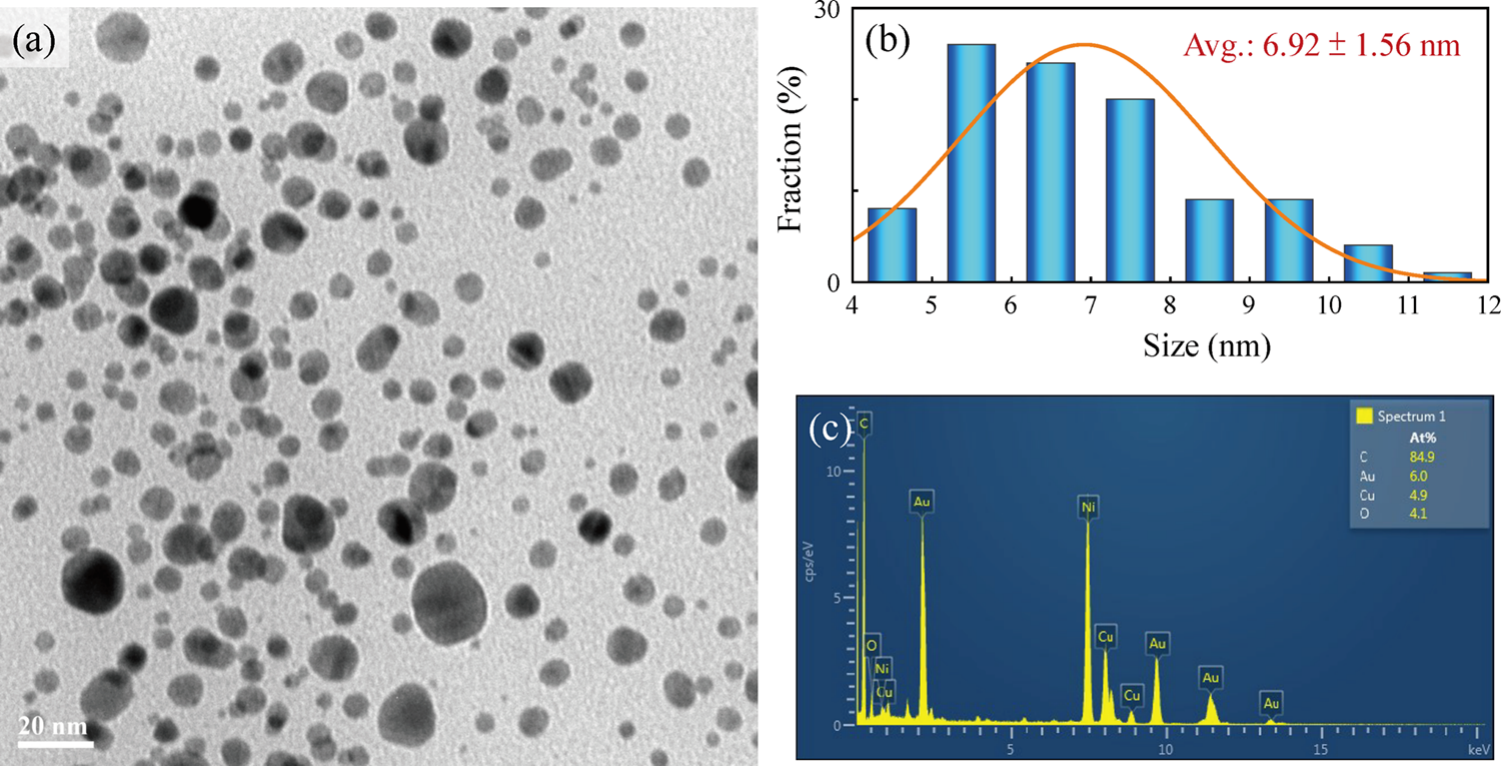
(a) TEM image, (b) particle size histogram, and (c) corresponding EDX profile of the TA-CuAu nanoparticles.

UV–vis absorption spectrometry is a main technique used for confirming the formation of the CuAu bimetallic nanoparticles. Under the optimal synthetic conditions, the spectrum of the TA-CuAu nanoparticles exhibited a characteristic absorption peak at approximately 526 nm, owing to the surface plasmon resonance (SPR) of the collective oscillations of the free electrons in the metal nanoparticles (Figure S4a). The size and shape of the formed nanoparticles were examined by using TEM analysis. Based on the TEM image presented in Figure 1, the TA-CuAu nanoparticles are well dispersed and nearly spherical. The average size of the TA-CuAu nanoparticles was calculated as 6.9 ± 1.6 nm. Furthermore, the chemical composition of the TA-CuAu nanoparticles was determined via an energy dispersive X-ray (EDX) analysis. The EDX spectrum (Figure 1c) comprised strong peaks of C, O, Au, and Cu, which were the constituent elements of the organic layer surrounding the nanoparticles. The dominant element with the highest weight percent in the TA-CuAu nanoparticles was C (84.9%), which was followed by Au (6.0%), Cu (4.9%), and O (4.1%). The crystal structure of the TA-CuAu nanoparticles was investigated by using XRD analysis. The XRD pattern of the TA-CuAu nanoparticles is presented in Figure S5a. Well-defined diffraction peaks were observed at 2θ values of 38.4°, 44.5°, 64.8°, 77.9°, and 81.8°, corresponding to the (111), (200), (220), (311), and (222) planes of the face-centered cubic facets of Au, respectively.^31^ The sharp and narrow diffraction peaks indicate the highly crystalline characteristics of the TA-CuAu nanoparticles.

Various functional groups responsible for the stabilization of the CuAu nanoparticles were identified via FTIR analysis based on their absorption positions. As depicted in Figure S6, the FTIR spectra of TA and the TA-CuAu nanoparticles are similar, indicating the successful stabilization of the synthesized CuAu nanoparticles by TA. Considering the pure TA spectrum, the broad peak located between 3600 and 3000 cm^–1^ corresponds to the O–H stretching vibrations of the hydroxyl groups of the TA molecules.^32^ The peak at 1718 cm^–1^ is attributed to carbonyl (C=O) stretching, and the peaks at 1612 and 1534 cm^–1^ are assigned to aromatic C=C stretching.^33^ The peaks at 1446 and 1322 cm^–1^ correspond to the deformation of −C–C– in phenolic and phenol groups, respectively.^34^ Additionally, the peaks at 1201, 1089, and 1030 cm^–1^ correspond to the C–H bonds (in-plane deformation) in the benzene ring and C–O and C–H deformations, respectively.^35^ These findings are consistent with the FTIR results reported for TA in the previous literature,^33,34^ confirming the presence of numerous functional groups, such as −OH, −COOH, and Ph–OH, on the TA molecules. The FTIR spectrum of the TA-CuAu nanoparticles exhibited the characteristic absorption peaks of TA. The spectrum of the TA-CuAu nanoparticles exhibited several characteristic absorption peaks at the same positions as those in the TA spectrum. However, a change in peak intensities was observed. Therefore, the FTIR results indicate that the surface of the CuAu nanoparticles has been successfully capped by the TA molecules.

**Figure 2:**
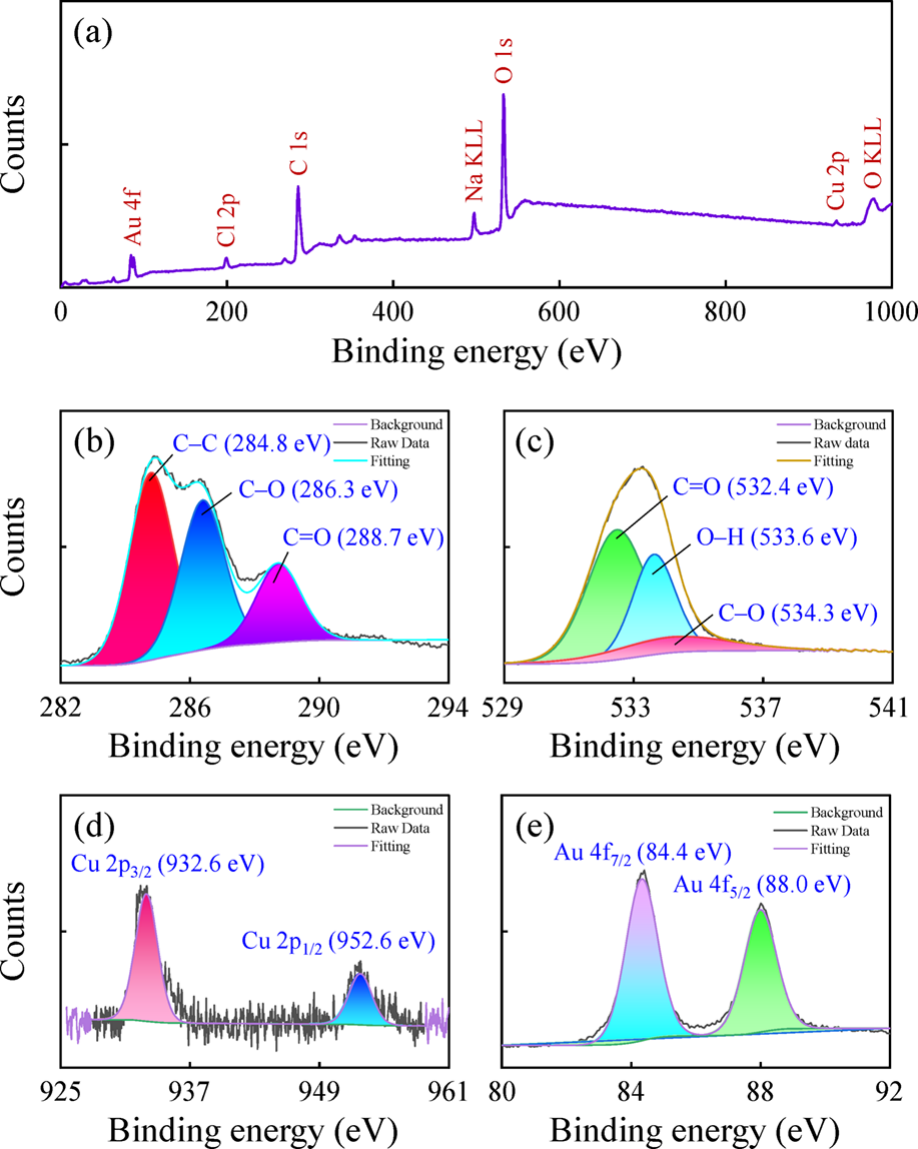
(a) XPS full survey spectra of TA-CuAu nanoparticles. High-resolution XPS spectra of (b) C 1s, (c) O 1s, (d) Cu 2p, and (e) Au 4f of the TA-CuAu nanoparticles.

XPS analysis was performed to study the elemental composition and chemical states of the TA-CuAu nanoparticles. As shown in Figure 2a, the peaks at 84.5, 285.5, 532.5, and 933.5 eV are ascribed to Au 4f, C 1s, O 1s, and Cu 2p, respectively. The deconvolution of the C 1s peak revealed constituent peaks at 284.8, 286.3, and 288.7 eV corresponding to C–C, C–O, and C=O, respectively (Figure 2b).^28^ Three characteristic peaks of C=O (532.4 eV), O–H (533.6 eV), and C–O (534.3 eV) were observed in the high-resolution O 1s spectrum (Figure 2c).^36^ The Cu 2p spectrum (Figure 2d) was fitted with two peaks at 932.6 (Cu 2p_3/2_) and 952.6 eV (Cu 2p_1/2_), indicating the presence of both Cu^0^ and Cu^+^.^37^ The Au 4f spectrum exhibited two notable peaks at 84.4 (Au 4f_7/2_) and 88.0 eV (Au 4f_5/2_) with a splitting energy of 3.6 eV (Figure 2e), corresponding to the zero oxidation state of Au.^38^ These findings further confirm the successful preparation of the TA-capped CuAu nanoparticles.

### 3.3 Stability of the TA-Capped CuAu Nanoparticles

To assess the stability of the TA-CuAu nanoparticles under various conditions, the effects of the buffer pH, NaCl concentration, and storage time on the absorption characteristics were investigated (Figure S7), which are important factors determining their practical applications. The as-prepared TA-CuAu nanoparticles were stable in Tris-HCl buffers with pH values of 5.0–9.0. The absorption spectra remained almost unchanged at NaCl concentrations ranging from 0 to 50 mM. Zeta potential measurements (−30.1 mV) revealed the formation of a highly stable colloidal solution of the TA-CuAu nanoparticles (Figure S7c). However, the aggregation of nanoparticles over time could strongly affect their catalytic activity. Therefore, the solution was stored in a refrigerator at 4 °C. No significant changes were observed in the optical spectra of the TA-CuAu nanoparticles during the initial 30 days, confirming the high stability of the as-synthesized TA-CuAu nanoparticles (Figure S7d).

### 3.4 Catalytic Performance of the Catalysts

The catalytic performance of the as-synthesized TA-CuAu nanoparticles was assessed through the degradation of 4-NP and RB in the presence of NaBH_4_. The catalytic mechanism for the degradation of 4-NP and RB using catalysts in the presence of NaBH_4_ has been well documented.^39−42^ The catalytic process comprises three steps: (1) the target molecules (4-NP and RB) adsorb on the surface of the CuAu nanoparticles; (2) electron transport occurs between the target molecules and NaBH_4_, resulting in the formation of the degraded products of the target molecules; and (3) the degraded products of the target molecules are subsequently released from the surface of the CuAu nanoparticles, the adsorption process of the target molecules resumes, and the surface is ready for a new cycle of the degradation of the target molecules. In this study, to prevent the overlap of the SPR peaks of the TA-CuAu nanoparticles and the main spectral peaks of 4-NP and RB, the TA-CuAu nanoparticles were used in small amounts and were treated with NaBH_4_. The progress of dye degradation and transformation of the colored reaction solution to a colorless solution owing to dye degradation was evaluated using UV–vis spectroscopy. The characteristic peaks of 4-NP and RB were recorded at 400 and 554 nm, respectively. The characteristic absorption of 4-NP and RB remained almost unchanged in the absence of the TA-CuAu nanoparticles, indicating that the progress of the degradation reaction was substantially slow in the sole presence of NaBH_4_ (Figure S8).

**Figure 3:**
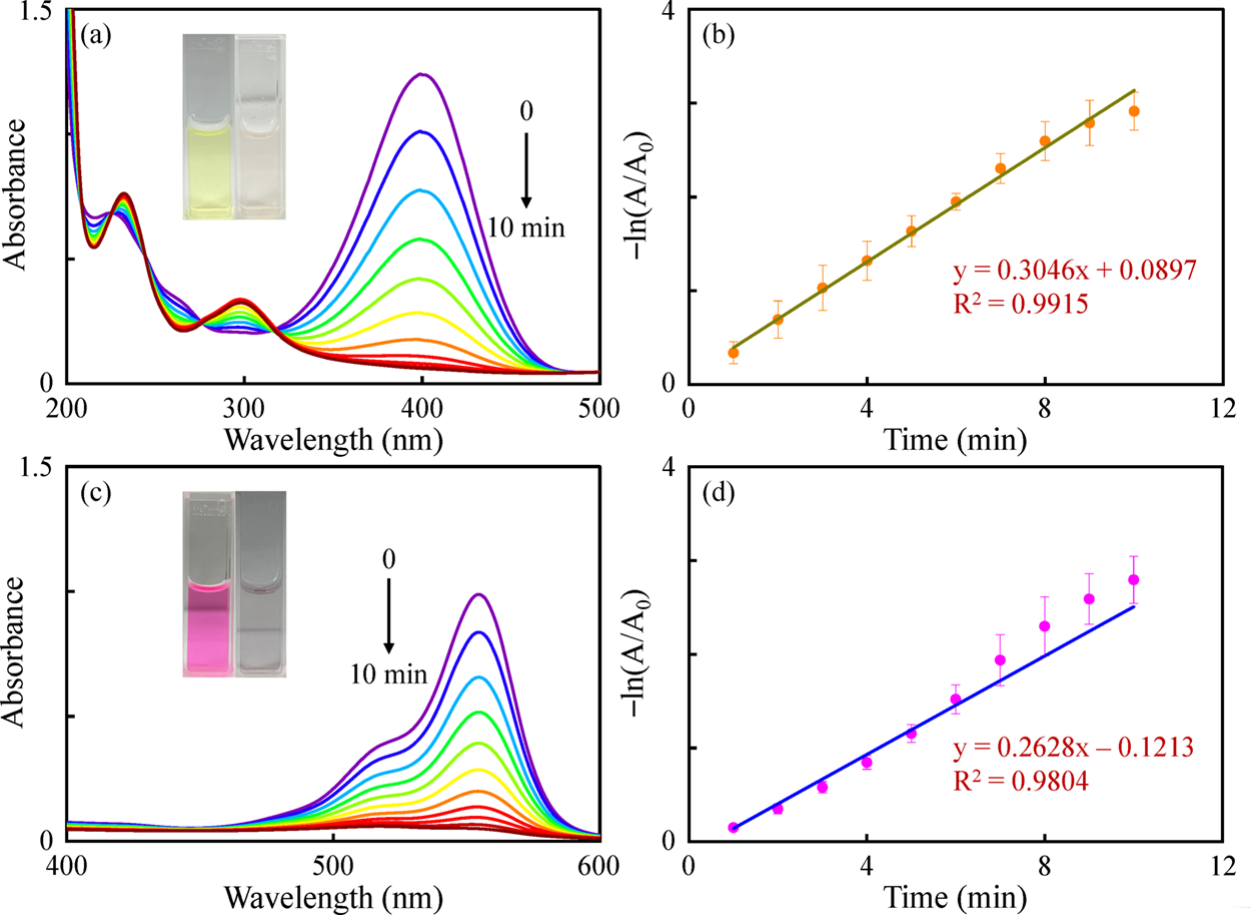
Time-dependent UV–vis absorption spectra for the degradation of (a) 4-NP and (c) RB using the TA-CuAu nanoparticles in the presence of NaBH_4_. Plots of −ln(*A*/*A*_0_) versus reaction time for the TA-CuAu nanoparticles with the absorbance of (b) 4-NP at 400 nm and (d) RB at 554 nm.

As shown in Figure 3, after the addition of the TA-CuAu nanoparticles, a rapid decrease in the absorbance and a change in the color of the 4-NP and RB solution are observed within 10 min, providing significant evidence for the high catalytic degradation ability of the TA-CuAu nanoparticles. As the concentration of NaBH_4_ used was often significantly higher than those of 4-NP and RB, reaction kinetics can be determined using the pseudo-first-order equation:^43^
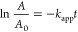


where *A*_0_ is the absorbance at the initial time; *A* is the absorbance at the final time; *t* is the degradation time; and *k*_app_ is the apparent rate constant (min^–1^). As can be seen from Figure 3, the −ln(*A*/*A*_0_) versus time plot exhibits a linear relationship, with *R*^2^ values of 0.9915 and 0.9804 for 4-NP and RB, respectively, further confirming the pseudo-first-order kinetics of catalytic degradation. The calculated rate constants for the degradation of 4-NP and RB by the TA-CuAu nanoparticles were 0.3046 and 0.2628 min^–1^, respectively. Compared with the TA-Cu and TA-Au nanoparticles, the bimetallic TA-CuAu nanoparticles exhibited the highest catalytic activity (Figure S9). The activity parameter *k′*, which is defined as the ratio *k*_app_ to the total mass of the catalyst, was introduced. The *k*′** value of the TA-CuAu nanoparticles is the highest of all listed catalysts (Table 1). The comparison results presented in Table 1 indicate that the TA-CuAu nanoparticles possess excellent catalytic activity comparable to or higher than that of other nanomaterial-based catalysts.

**Table 1:** Comparative Analysis of the Catalytic Activity of the As-Synthesized TA-CuAu Nanoparticles and Previously Reported Nanomaterial-Based Catalysts for the Degradation of 4-NP and RB^a^

dye	catalyst	amount (mg)	time (min)	*k*_app_ (min^–1^)	*k'* [*k*_app_/catalyst amount (min^–1^/mg)]	ref
4-NP	PtRh ANMPs	0.02	20	0.2090	10.45	(44)
	Fe_3_O_4_@TA/Ag	2.00	1	2.6160	1.31	(45)
	Cu/MC/Fe_3_O_4_ NPs	0.10	19	1.1000	11.00	(46)
	Fe_3_O_4_@PS@Ag	2.00	3	0.5160	0.26	(47)
	AgNi@ZnO nanocomposites	0.30	2	0.8544	2.85	(48)
	TA-CuAu NPs	0.01	10	0.3046	30.46	this work
RB	PtRh ANMPs	0.02	20	0.3540	17.70	(44)
	Fe_3_O_4_@TA/Ag	2.00	1	3.0630	1.55	(45)
	Cu/MC	0.10	19	0.2300	2.30	(46)
	Fe_3_O_4_@PS@Ag	2.00	0.8	1.1220	0.56	(47)
	AgNi@ZnO nanocomposites	0.30	1.5	0.8280	2.76	(48)
	TA-CuAu NPs	0.01	10	0.2628	26.28	this work

a ANMPs: alloyed nanomultipods; MC: magnetic carbon; NPs: nanoparticles; PS: polystyrene; TA: tannic acid.

**Figure 4:**
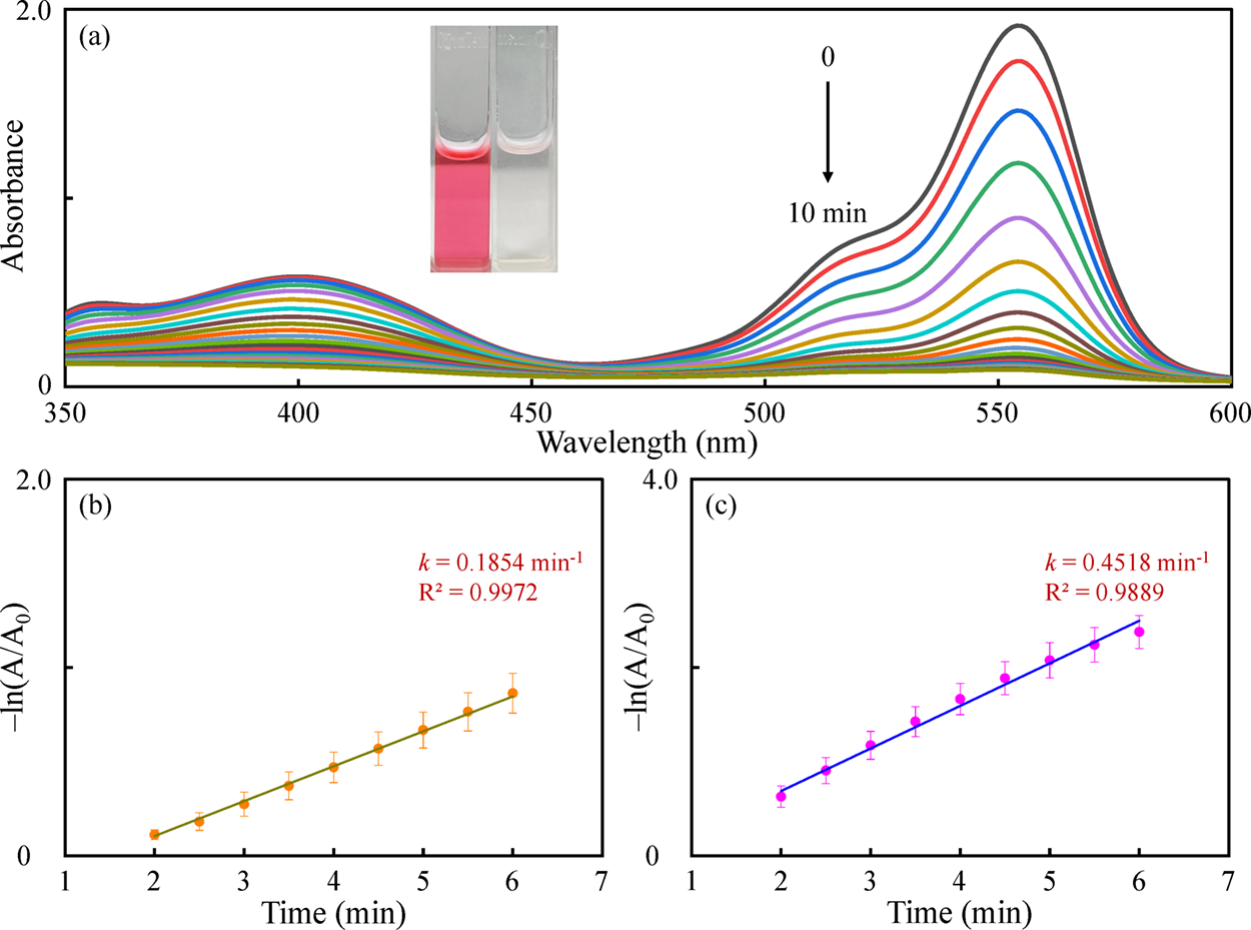
(a) Simultaneous degradation of 4-NP (0.25 mM) and RB (0.1 mM) by the TA-CuAu nanoparticles in the presence of NaBH_4_. Plots of −ln(*A*/*A*_0_) versus time for the absorbance at (b) 400 and (c) 554 nm for the degradation of 4-NP and RB, respectively.

To evaluate the catalytic activity of the TA-CuAu nanoparticles for the simultaneous degradation of 4-NP and RB, 4-NP (0.25 mM) and RB (0.1 mM) were mixed and degraded using the TA-CuAu nanoparticles in the presence of NaBH_4_. As can be seen from Figure 4, the TA-CuAu nanoparticles efficiently degrade the mixture of 4-NP and RB, as indicated by the colorless solution, and a decrease in the absorbance at 400 and 554 nm was observed for 4-NP and RB, respectively. The percentages of degradation were 84.46 and 93.58%, and the calculated rate constants were 0.1854 and 0.4518 min^–1^ for the simultaneous degradation of 4-NP and RB, respectively. The catalytic performance for the simultaneous degradation of 4-NP and RB with monometallic nanoparticles (TA-Cu and TA-Au nanoparticles) is shown in Figure S10, and only a few mixed dyes were degraded in 10 min. The results proved the synergistic effect in the bimetallic system.

Apart from the catalytic activity, the reusability and stability of the TA-CuAu nanoparticles are tested. After the reduction process, the TA-CuAu nanoparticles were separated from the reaction solution by centrifugation, and the TA-CuAu nanoparticles were again used five times for the reduction of another new 4-NP and RB solution. Figure S11 shows the stability of the TA-CuAu nanoparticles with a degradation efficiency greater than 86.92 and 92.28% for 4-NP and RB, respectively, to five cycles of reduction. As Figure S5b,c shows, no remarkable change is noticed for the XRD patterns after the five consecutive cycles in the cases of 4-NP and RB. This shows that the prepared TA-CuAu nanoparticles as catalysts can be used for practical catalytic applications.

### 3.5 Degradation of 4-Nitrophenol and Rhodamine B in Water Samples

To evaluate the practical viability and efficiency of the TA-CuAu nanoparticles, the degradation of 4-NP and RB was performed by using the tap and lake water samples. Initially, 4-NP and RB solutions were prepared using tap water and lake water samples. The degradation efficiencies for 4-NP and RB were calculated as 94.49 and 88.82% for tap water and 94.17 and 87.76% for lake water (Figures S12 and S13), respectively, using the TA-CuAu nanoparticles as catalysts with the addition of freshly prepared NaBH_4_. The calculated reaction rate was lower in the tap and lake water samples than in DI water.

## 4 Conclusions

In summary, a facile approach was adopted to prepare bimetallic CuAu nanoparticles via chemical reduction using TA as a stabilizing agent. The characteristic SPR band of the TA-CuAu nanoparticles in the absorption spectrum was observed at 526 nm, indicating their successful synthesis. Moreover, the TA-CuAu nanoparticles were applied as catalysts, and the degradation of 4-NP and RB to less toxic products was successfully achieved within 10 min in the presence of NaBH_4_ as a reducing agent, with *k*_app_ values of 0.3046 and 0.2628 min^–1^, respectively. The excellent performance of the as-synthesized catalysts in the simultaneous degradation of 4-NP and RB illustrates their application potential for degrading a mixture of various pollutants with a high catalytic efficiency. In addition, good recycling ability is shown by the TA-CuAu nanoparticles after five times the catalytic reduction of 4-NP and RB. Considering their high catalytic activity, the TA-CuAu nanoparticles are anticipated to be used in various applications, such as water-pollutant degradation.

## Supporting Information Available

Influence of various experimental parameters on the synthesis of TA-CuAu nanoparticles; characterization of the TA-CuAu, TA-Au, and TA-Cu nanoparticles; effect of pH, NaCl concentration, and storage time on the UV–vis spectra of the TA-CuAu nanoparticles; zeta potential measurements; catalytic degradation of 4-NP and RB in the absence of the TA-CuAu nanoparticles and NaBH_4_; reusability of the TA-CuAu nanoparticles; simultaneous degradation of 4-NP and RB by the TA-Cu and TA-Au nanoparticles; time-dependent UV–vis absorption spectra for the degradation of spiked 4-NP and RB in real samples by the TA-CuAu nanoparticles; and degradation percentages of the TA-CuAu nanoparticles toward 4-NP and RB in real samples (PDF)

## References

[ref1] Krishnan A.; Swarnalal A.; Das D.; Krishnan M.; Saji V. S.; Shibli S. M. A. A Review on Transition Metal Oxides Based Photocatalysts for Degradation of Synthetic Organic Pollutants. J. Environ. Sci. 2024, 139, 389–417. 10.1016/j.jes.2023.02.051. 38105064

[ref2] Balakrishnan A.; Gaware G. J.; Chinthala M. Heterojunction Photocatalysts for the Removal of Nitrophenol: A Systematic Review. Chemosphere 2023, 310, 136853 10.1016/j.chemosphere.2022.136853. 36243095

[ref3] Karbasi M.; Nikoomanzari E.; Hosseini R.; Bahramian H.; Chaharmahali R.; Giannakis S.; Kaseem M.; Fattah-alhosseini A. A Review on Plasma Electrolytic Oxidation Coatings for Organic Pollutant Degradation: How to Prepare Them and What to Expect of Them?. J. Environ. Chem. Eng. 2023, 11 (3), 110027 10.1016/j.jece.2023.110027.

[ref4] Bisaria K.; Sinha S.; Singh R.; Iqbal H. M. N. Recent Advances in Structural Modifications of Photo-Catalysts for Organic Pollutants Degradation – A Comprehensive Review. Chemosphere 2021, 284, 131263 10.1016/j.chemosphere.2021.131263. 34198058

[ref5] Zhao X.; Chang Y.; Chen W.-J.; Wu Q.; Pan X.; Chen K.; Weng B. Recent Progress in Pd-Based Nanocatalysts for Selective Hydrogenation. ACS Omega 2022, 7 (1), 17–31. 10.1021/acsomega.1c06244. 35036674 PMC8756445

[ref6] Ndolomingo M. J.; Bingwa N.; Meijboom R. Review of Supported Metal Nanoparticles: Synthesis Methodologies, Advantages and Application as Catalysts. J. Mater. Sci. 2020, 55 (15), 6195–6241. 10.1007/s10853-020-04415-x.

[ref7] Gao C.; Lyu F.; Yin Y. Encapsulated Metal Nanoparticles for Catalysis. Chem. Rev. 2021, 121 (2), 834–881. 10.1021/acs.chemrev.0c00237. 32585087

[ref8] Guo Y.; Wen M.; Li G.; An T. Recent Advances in VOC Elimination by Catalytic Oxidation Technology onto Various Nanoparticles Catalysts: A Critical Review. Appl. Catal., B 2021, 281, 119447 10.1016/j.apcatb.2020.119447.

[ref9] Doan V.-D.; Pham Q.-H.; Huynh B.-A.; Nguyen T.-L.-H.; Nguyen A.-T.; Nguyen T.-D. Kinetic Analysis of Nitrophenol Reduction and Colourimetric Detection of Hydrogen Peroxide Based on Gold Nanoparticles Catalyst Biosynthesised from *Cynomorium Songaricum*. J. Environ. Chem. Eng. 2021, 9 (6), 106590 10.1016/j.jece.2021.106590.

[ref10] Ambreen J.; Al-Harbi F. F.; Sakhawat H.; Ajmal M.; Naeem H.; Farooqi Z. H.; Batool N.; Siddiq M. Fabrication of Poly (*N*-Vinylcaprolactam-co-Acrylic Acid)-Silver Nanoparticles Composite Microgel with Substantial Potential of Hydrogen Peroxide Sensing and Catalyzing the Reduction of Water Pollutants. J. Mol. Liq. 2022, 355, 118931 10.1016/j.molliq.2022.118931.

[ref11] Nguyen T. H. A.; Nguyen V.-C.; Phan T. N. H.; Le V. T.; Vasseghian Y.; Trubitsyn M. A.; Nguyen A.-T.; Chau T. P.; Doan V.-D. Novel Biogenic Silver and Gold Nanoparticles for Multifunctional Applications: Green Synthesis, Catalytic and Antibacterial Activity, and Colorimetric Detection of Fe(III) Ions. Chemosphere 2022, 287, 132271 10.1016/j.chemosphere.2021.132271. 34547560

[ref12] Begum R.; Ahmad G.; Najeeb J.; Wu W.; Irfan A.; Azam M.; Nisar J.; Farooqi Z. H. Stabilization of Silver Nanoparticles in Crosslinked Polymer Colloids through Chelation for Catalytic Degradation of p-Nitroaniline in Aqueous Medium. Chem. Phys. Lett. 2021, 763, 138263 10.1016/j.cplett.2020.138263.

[ref13] Liang C.; Cheong J. Y.; Sitaru G.; Rosenfeldt S.; Schenk A. S.; Gekle S.; Kim I.-D.; Greiner A. Size-Dependent Catalytic Behavior of Gold Nanoparticles. Adv. Mater. Interfaces 2022, 9 (4), 2100867 10.1002/admi.202100867.

[ref14] Garg N.; Bera S.; Rastogi L.; Ballal A.; Balaramakrishna M. V. Synthesis and Characterization of L-Asparagine Stabilised Gold Nanoparticles: Catalyst for Degradation of Organic Dyes. Spectrochim. Acta A Mol. Biomol. Spectrosc. 2020, 232, 118126 10.1016/j.saa.2020.118126. 32062492

[ref15] Wang W.-Y.; Chiu C.-L.; Hu C.-C.; Chiu T.-C. Ag Nanoparticles Decorated by Gallic Acid as a Colorimetric Sensor for the Detection of Cartap Pesticide. ACS Appl. Nano Mater. 2023, 6 (16), 15324–15329. 10.1021/acsanm.3c03402.

[ref16] Xiao W.-Z.; Xiao L.-P.; Yang Y.-Q.; Xu Q.; He W.-Q.; Zhang J.; Wang R.-Y.; Zhao X.; Zhai S.-R.; Sun R.-C. Fully Exposed Silver Nanoparticles Stabilized on pH-Responsive Lignin-Reactors for Enhanced 4-Nitrophenol Reduction. J. Environ. Chem. Eng. 2022, 10 (3), 107945 10.1016/j.jece.2022.107945.

[ref17] Naseem K.; Ali F.; Tahir M. H.; Afaq M.; Yasir H. M.; Ahmed K.; Aljuwayid A. m.; Habila M. A. Investigation of Catalytic Potential of Sodium Dodecyl Sulfate Stabilized Silver Nanoparticles for the Degradation of Methyl Orange Dye. J. Mol. Struct. 2022, 1262, 132996 10.1016/j.molstruc.2022.132996.

[ref18] Mejía Y. R.; Bogireddy N. K. R. Reduction of 4-Nitrophenol Using Green-Fabricated Metal Nanoparticles. RSC Adv. 2022, 12 (29), 18661–18675. 10.1039/D2RA02663E. 35873318 PMC9228544

[ref19] Zhang X.-Q.; Shen R.-F.; Guo X.-J.; Yan X.; Chen Y.; Hu J.-T.; Lang W.-Z Bimetallic Ag-Cu Nanoparticles Anchored on Polypropylene (PP) Nonwoven Fabrics: Superb Catalytic Efficiency and Stability in 4-Nitrophenol Reduction. Chem. Eng. J. 2021, 408, 128018 10.1016/j.cej.2020.128018.

[ref20] Ouyang L.; Noël V.; Courty A.; Campagne J.-M.; Ouali A.; Vrancken E. Copper Nanoparticles with a Tunable Size: Implications for Plasmonic Catalysis. ACS Appl. Nano Mater. 2022, 5 (2), 2839–2847. 10.1021/acsanm.2c00016.

[ref21] Yin J.; Shan S.; Yang L.; Mott D.; Malis O.; Petkov V.; Cai F.; Ng M. S.; Luo J.; Chen B. H.; Engelhard M.; Zhong C.-J. Gold–Copper Nanoparticles: Nanostructural Evolution and Bifunctional Catalytic Sites. Chem. Mater. 2012, 24 (24), 4662–4674. 10.1021/cm302097c.

[ref22] Hofmann D. M.; Fairbrother D. H.; Hamers R. J.; Murphy C. J. Two-Phase Synthesis of Gold–Copper Bimetallic Nanoparticles of Tunable Composition: Toward Optimized Catalytic CO_2_ Reduction. ACS Appl. Nano Mater. 2019, 2 (6), 3989–3998. 10.1021/acsanm.9b00904.

[ref23] Boeva O.; Kudinova E.; Vorakso I.; Zhavoronkova K.; Antonov A. Bimetallic Gold-Copper Nanoparticles in the Catalytic Reaction of Deuterium-Hydrogen Exchange: A Synergistic Effect. Int. J. Hydrog. Energy 2022, 47 (7), 4759–4765. 10.1016/j.ijhydene.2021.11.078.

[ref24] Ahmad M.; Nawaz T.; Assiri M. A.; Hussain R.; Hussain I.; Imran M.; Ali S.; Wu Z. Fabrication of Bimetallic Cu–Ag Nanoparticle-Decorated Poly(cyclotriphosphazene-co-4,4′-sulfonyldiphenol) and Its Enhanced Catalytic Activity for the Deduction of 4-Nitrophenol. ACS Omega 2022, 7 (8), 7096–7102. 10.1021/acsomega.1c06786. 35252700 PMC8892640

[ref25] Torkamani F.; Azizian S. Green and Simple Synthesis of Ag Nanoparticles Loaded onto Cellulosic Fiber as Efficient and Low-Cost Catalyst for Reduction of 4-Nitrophenol. J. Mol. Liq. 2016, 214, 270–275. 10.1016/j.molliq.2015.12.071.

[ref26] Xu X.; Li M.; Yang L.; Hu B. Remarkably and Stable Catalytic Activity in Reduction of 4-Nitrophenol by Sodium Sesquicarbonate-Supporting Fe_2_O_3_@Pt. RSC Adv. 2023, 13 (20), 13556–13563. 10.1039/D3RA01930F. 37152584 PMC10155080

[ref27] Liu F.; Liu X.; Chen F.; Fu Q. Tannic Acid: A Green and Efficient Stabilizer of Au, Ag, Cu and Pd Nanoparticles for the 4-Nitrophenol Reduction, Suzuki–Miyaura Coupling Reactions and Click Reactions in Aqueous Solution. J. Colloid Interface Sci. 2021, 604, 281–291. 10.1016/j.jcis.2021.07.015. 34271489

[ref28] Liu S.; Yin S.; Jiao S.; Zhang H.; Wang Z.; Xu Y.; Li X.; Wang L.; Wang H. Au Nanowire Modified with Tannic Acid for Enhanced Electrochemical Synthesis of Ammonia. Mater. Today Energy 2021, 21, 100828 10.1016/j.mtener.2021.100828.

[ref29] Savin R.; Benzaamia N.-O.; Njel C.; Pronkin S.; Blanck C.; Schmutz M.; Boulmedais F. Nanohybrid Biosensor Based on Mussel-Inspired Electro-cross-Linking of Tannic Acid Capped Gold Nanoparticles and Enzymes. Mater. Adv. 2022, 3 (4), 2222–2233. 10.1039/D1MA01193F.

[ref30] Zhang J.; Xu X.; Yang C.; Yang F.; Yang X. Colorimetric Iodide Recognition and Sensing by Citrate-Stabilized Core/Shell Cu@Au Nanoparticles. Anal. Chem. 2011, 83 (10), 3911–3917. 10.1021/ac200480r. 21449559

[ref31] Ren Y.; Rao R.; Bhusal S.; Varshney V.; Kedziora G.; Wheeler R.; Kang Y.; Roy A.; Nepal D. Hierarchical Assembly of Gold Nanoparticles on Graphene Nanoplatelets by Spontaneous Reduction: Implications for Smart Composites and Biosensing. ACS Appl. Nano Mater. 2020, 3 (9), 8753–8762. 10.1021/acsanm.0c01555.

[ref32] Guo Q.; Li S.; Du G.; Chen H.; Yan X.; Chang S.; Yue T.; Yuan Y. Formulation and Characterization of Microcapsules Encapsulating Carvacrol Using Complex Coacervation Crosslinked with Tannic Acid. LWT 2022, 165, 113683 10.1016/j.lwt.2022.113683.

[ref33] Liang X.; Cao K.; Li W.; Li X.; McClements D. J.; Hu K. Tannic Acid-Fortified Zein-pectin Nanoparticles: Stability, Properties, Antioxidant Activity, and *In Vitro* Digestion. Food Res. Int. 2021, 145, 110425 10.1016/j.foodres.2021.110425. 34112427

[ref34] Li D.; Liu C.; Liu Y.; Chen X.; Wu W.; Li F.; Tian J.; Dang Z. Tannic Acid as an Eco-Friendly Natural Passivator for the Inhibition of Pyrite Oxidation to Prevent Acid Mine Drainage at the Source. Appl. Surf. Sci. 2022, 591, 153172 10.1016/j.apsusc.2022.153172.

[ref35] Lee H.; Su Y.-C.; Tang H.-H.; Lee Y.-S.; Lee J.-Y.; Hu C.-C.; Chiu T.-C. One-Pot Hydrothermal Synthesis of Carbon Dots as Fluorescent Probes for the Determination of Mercuric and Hypochlorite Ions. Nanomaterials 2021, 11 (7), 1831 10.3390/nano11071831. 34361216 PMC8308378

[ref36] Xiang L.; Liu L.-L.; Yuan R.; Chai Y.-Q. Aggregation-Induced Electrochemiluminescence of Copper Nanoclusters by Regulating Valence State Ratio of Cu(I)/Cu(0) for Ultrasensitive Detection of MicroRNA. Anal. Chem. 2023, 95 (9), 4454–4460. 10.1021/acs.analchem.2c05029. 36880263

[ref37] Xiong L. L.; Huang R.; Chai H. H.; Yu L.; Li C. M. Facile Synthesis of Fe_3_O_4_@Tannic Acid@Au Nanocomposites as a Catalyst for 4-Nitrophenol and Methylene Blue Removal. ACS Omega 2020, 5 (33), 20903–20911. 10.1021/acsomega.0c02347. 32875225 PMC7450606

[ref38] Shi X.; Huang C.; Zheng Z.; Zhong B.; Ding G.; Li J.; You L.; Wang S. Preparation of Magnetically Recoverable MPCTP-Ag Composite Nanoparticles and Their Application as High-Performance Catalysts. Langmuir 2021, 37 (34), 10249–10258. 10.1021/acs.langmuir.1c00944. 34415769

[ref39] Saravanakumar K.; Priya V. S.; Balakumar V.; Prabavathi S. L.; Muthuraj V. Noble Metal Nanoparticles (M_x_ = Ag, Au, Pd) Decorated Graphitic Carbon Nitride Nanosheets for Ultrafast Catalytic Reduction of Anthropogenic Pollutant, 4-Nitrophenol. Environ. Res. 2022, 212, 113185 10.1016/j.envres.2022.113185. 35395238

[ref40] Khan S. A.; Sohni S.; Akhtar K.; Bakhsh E. M.; Nawaz T.; Khan S. B. Lignocellulose Biomatrix Zero-Valent Cobalt Nanoparticles: A Dip-Catalyst for Organic Pollutants Degradation. Ind. Crops Prod. 2023, 198, 116694 10.1016/j.indcrop.2023.116694.

[ref41] Ali F.; Mehmood S.; Ashraf A.; Saleem A.; Younas U.; Ahmad A.; Bhatti M. P.; Eldesoky G. E.; Aljuwayid A. M.; Habila M. A.; Bokhari A.; Mubashir M.; Chuah L. F.; Chong J. W. R.; Show P. L. Ag–Cu Embedded SDS Nanoparticles for Efficient Removal of Toxic Organic Dyes from Water Medium. Ind. Eng. Chem. Res. 2023, 62 (11), 4765–4777. 10.1021/acs.iecr.2c03460.

[ref42] Shao Y.-C.; Hsieh M.-M.; Liu C. C.; Wang W.-Y.; Xue P.-H.; Hu C.-C.; Chiu T.-C. Enhanced Catalytic Activity of Tannic Acid Functionalized Gold Nanorods toward the Reduction of 4-Nitrophenol. J. Mol. Struct. 2024, 1303, 137645 10.1016/j.molstruc.2024.137645.

[ref43] Najafi M.; Azizian S. Catalytic Reduction of 4-Nitrophenol on the Surface of Copper/Copper Oxide Nanoparticles: A Kinetics Study. Appl. Nanosci. 2020, 10 (10), 3827–3837. 10.1007/s13204-020-01485-w.

[ref44] Yan Q.; Wang X.-Y.; Feng J.-J.; Mei L.-P.; Wang A.-J. Simple Fabrication of Bimetallic Platinum-Rhodium Alloyed Nano-Multipods: A Highly Effective and Recyclable Catalyst for Reduction of 4-Nitrophenol and Rhodamine B. J. Colloid Interface Sci. 2021, 582, 701–710. 10.1016/j.jcis.2020.08.062. 32911415

[ref45] Veisi H.; Moradi S. B.; Saljooqi A.; Safarimehr P. Silver Nanoparticle-Decorated on Tannic Acid-Modified Magnetite Nanoparticles (Fe_3_O_4_@TA/Ag) for Highly Active Catalytic Reduction of 4-Nitrophenol, Rhodamine B and Methylene Blue. Mater. Sci. Eng., C 2019, 100, 445–452. 10.1016/j.msec.2019.03.036. 30948080

[ref46] Xu P.; Cen C.; Zheng M.; Wang Y.; Wu Z.; Teng Z. A Facile Electrostatic Droplets Assisted Synthesis of Copper Nanoparticles Embedded Magnetic Carbon Microspheres for Highly Effective Catalytic Reduction of 4-Nitrophenol and Rhodamine B. Mater. Chem. Phys. 2020, 253, 123444 10.1016/j.matchemphys.2020.123444.

[ref47] Wang Y.; Gao P.; Wei Y.; Jin Y.; Sun S.; Wang Z.; Jiang Y. Silver Nanoparticles Decorated Magnetic Polymer Composites (Fe_3_O_4_@PS@Ag) as Highly Efficient Reusable Catalyst for the Degradation of 4-Nitrophenol and Organic Dyes. J. Environ. Manag. 2021, 278, 111473 10.1016/j.jenvman.2020.111473. 33120097

[ref48] Sachi; Singh A. P.; Thirumal M. Fabrication of AgNi Nano-Alloy-Decorated ZnO Nanocomposites as an Efficient and Novel Hybrid Catalyst to Degrade Noxious Organic Pollutants. ACS Omega 2021, 6 (50), 34771–34782. 10.1021/acsomega.1c05266. 34963960 PMC8697397

