# Supplementary material for: Tannic Acid-Decorated Bimetallic Copper–Gold Nanoparticles with High Catalytic Activity for the Degradation of 4-Nitrophenol and Rhodamine B
Source: ACS Omega. 2024 Jun 3;9(23):24970–7. doi: 10.1021/acsomega.4c02036 (PMC11171089; doi:10.1021/acsomega.4c02036)
Supplement: Supplementary file 1 — ao4c02036_si_001.pdf [file ao4c02036_si_001.pdf]

Supporting Information (SI)

Tannic Acid Decorated Bimetallic Copper–Gold Nanoparticles with High Catalytic Activity for the Degradation of 4-Nitrophenol and Rhodamine B

Cheng-Chih Liu, Wei-Yu Wang, Cho-Chun Hu, and Tai-Chia Chiu*

Department of Applied Science, National Taitung University, 369, Section 2, University Road, Taitung 950309, Taiwan

Correspondence:
Professor Tai-Chia Chiu, Department of Applied Science, National Taitung University, 369, Section 2, University Road, Taitung 950309, Taiwan
Tel.: 886-89-517990; fax: 886-89-518108
E-mail: tcchiu@nttu.edu.tw

E-mail addresses: jpjpcalvkss@gmail.com (C-C. Liu), xx38084013@gmail.com (W.-Y. Wang), cchu@nttu.edu.tw (C.-C. Hu)

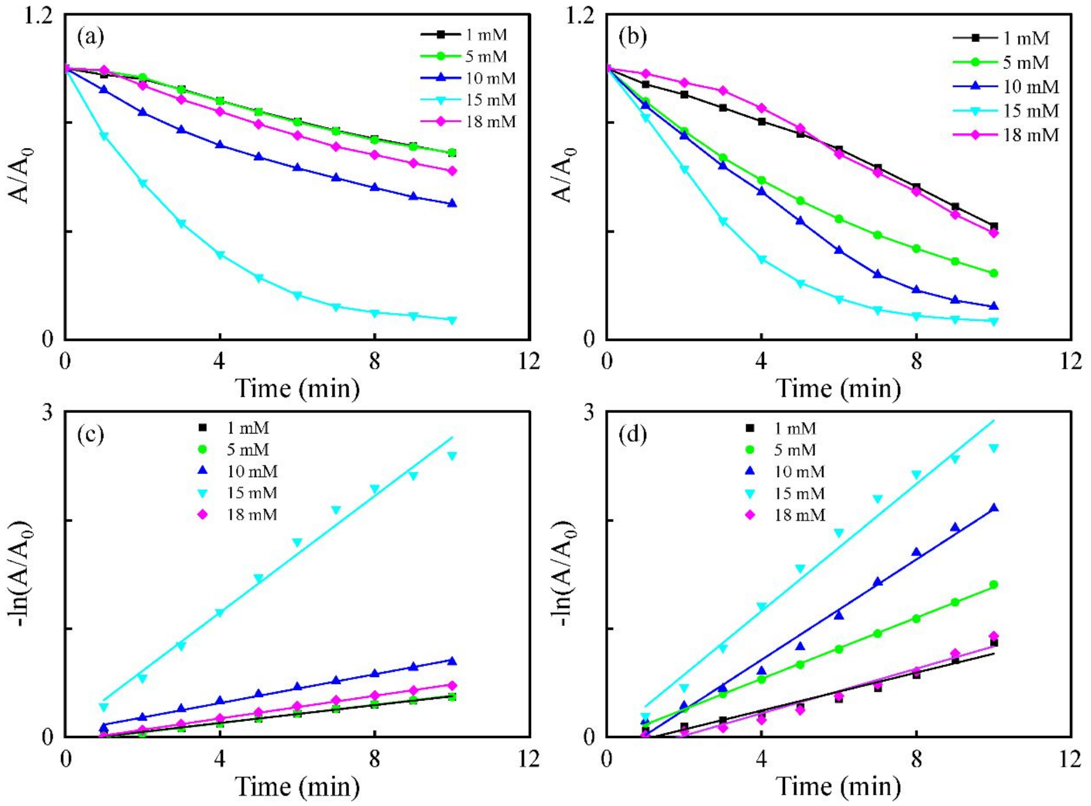

Figure S1. Catalytic degradation performances of (a) 4-NP and (b) RB by the TA-CuAu nanoparticles with various initial $NaAuCl_4$ concentrations for the synthesis of the TA-CuAu nanoparticles. Pseudo-first order kinetic linear simulation curves for (c) 4-NP and (d) RB.

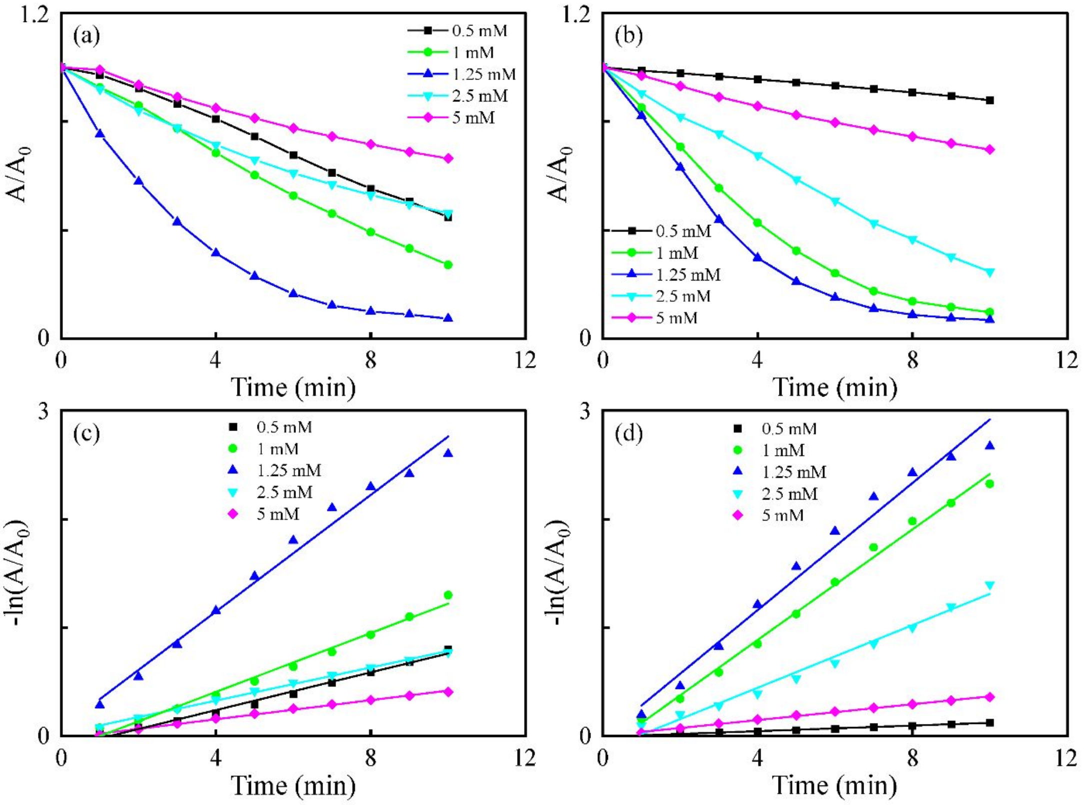

Figure S2. Catalytic degradation performances of (a) 4-NP and (b) RB by the TA-CuAu nanoparticles with various initial TA concentrations for the synthesis of the TA-CuAu nanoparticles. Pseudo-first order kinetic linear simulation curves for (c) 4-NP and (d) RB.

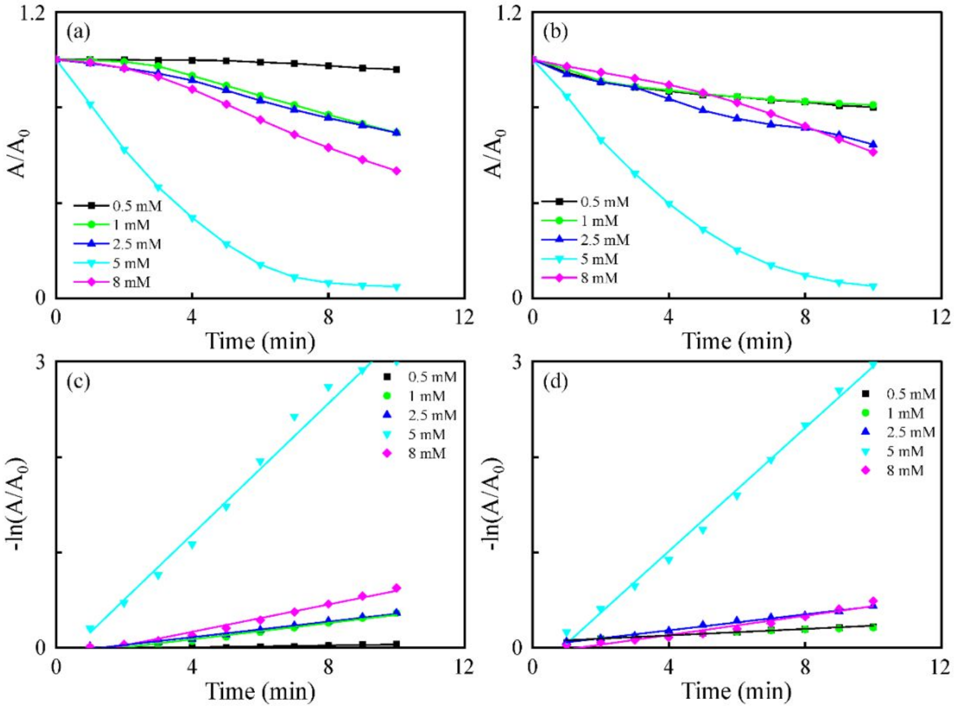

Figure S3. Catalytic degradation performances of (a) 4-NP and (b) RB by the TA-CuAu nanoparticles with various initial $NaBH_4$ concentrations for the synthesis of the TA-CuAu nanoparticles. Pseudo-first order kinetic linear simulation curves for (c) 4-NP and (d) RB.

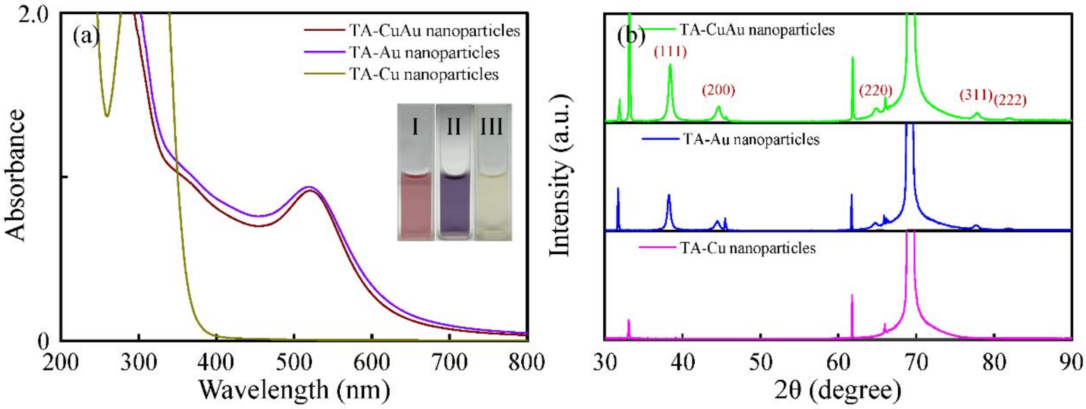

Figure S4. (a) UV–Vis spectra and (b) XRD patterns of TA-CuAu, TA-Au, and TA-Cu nanoparticles. Inset: A photograph of (I) TA-CuAu, (II) TA-Au, and (III) TA-Cu nanoparticles.

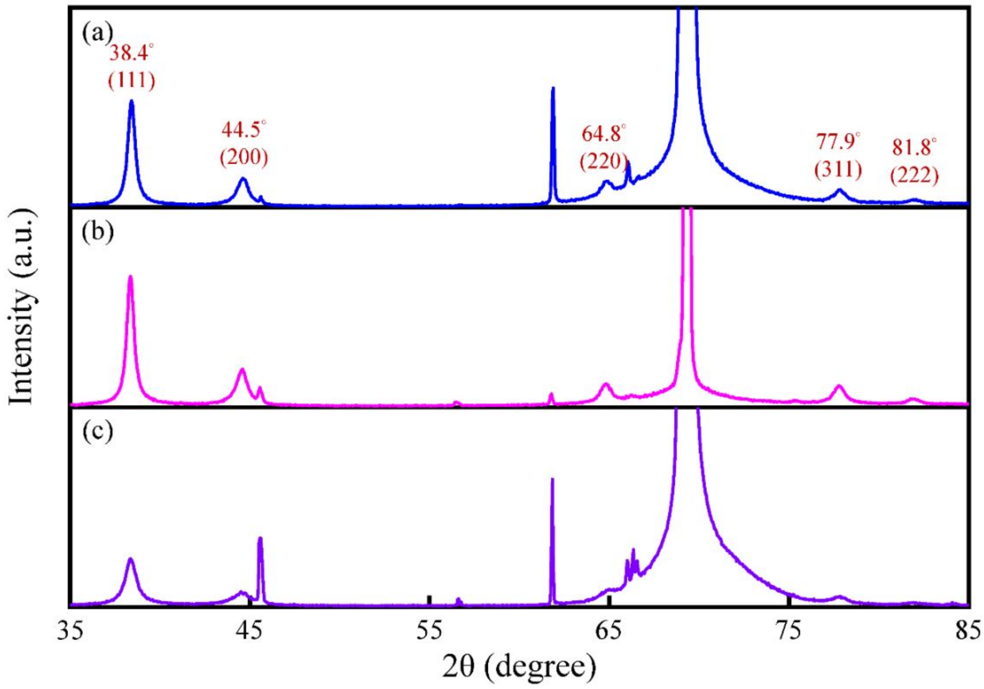

Figure S5. XRD patterns of (a) the TA-CuAu nanoparticles and recycled TA-CuAu nanoparticles after five reduction cycles of (b) 4-NP and (c) RB.

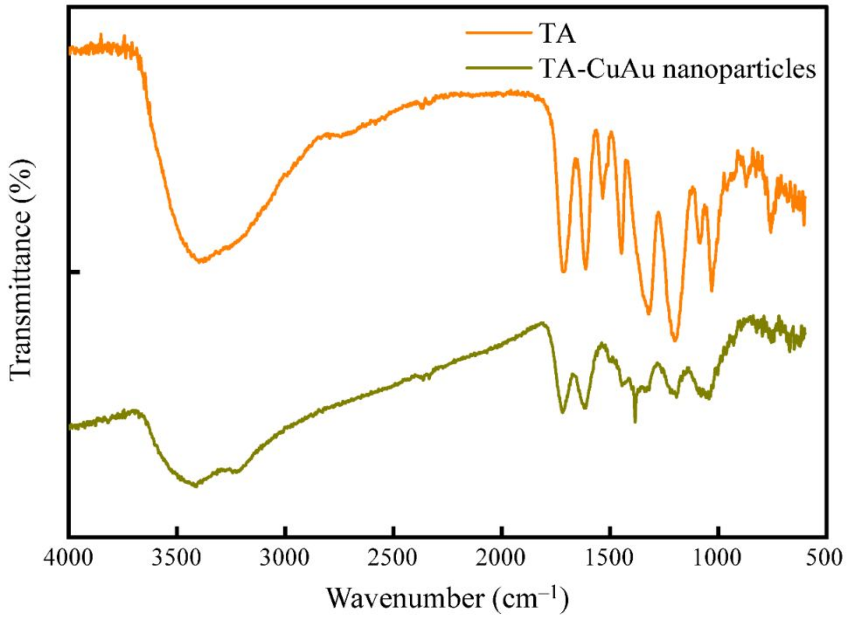

Figure S6. FTIR spectra of TA and the TA-CuAu nanoparticles.

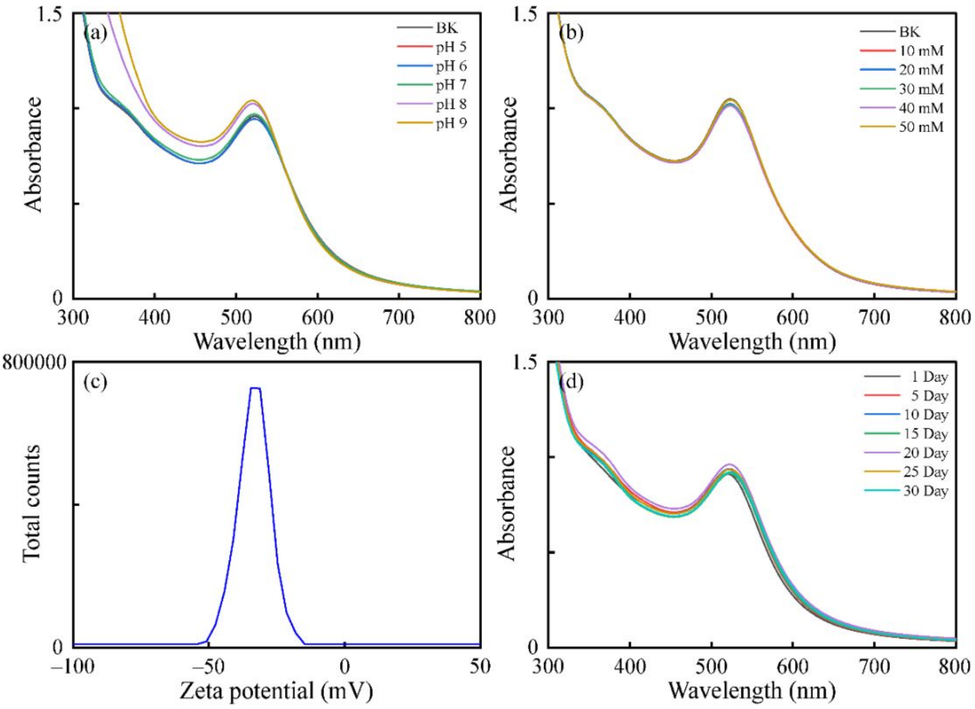

Figure S7. Effect of (a) buffer pH and (b) NaCl concentration on the UV–Vis spectra of the TA-CuAu nanoparticles. (c) Zeta potential measurements of the TA-CuAu nanoparticles. Effect of (d) storage time on the UV–Vis spectra of the TA-CuAu nanoparticles.

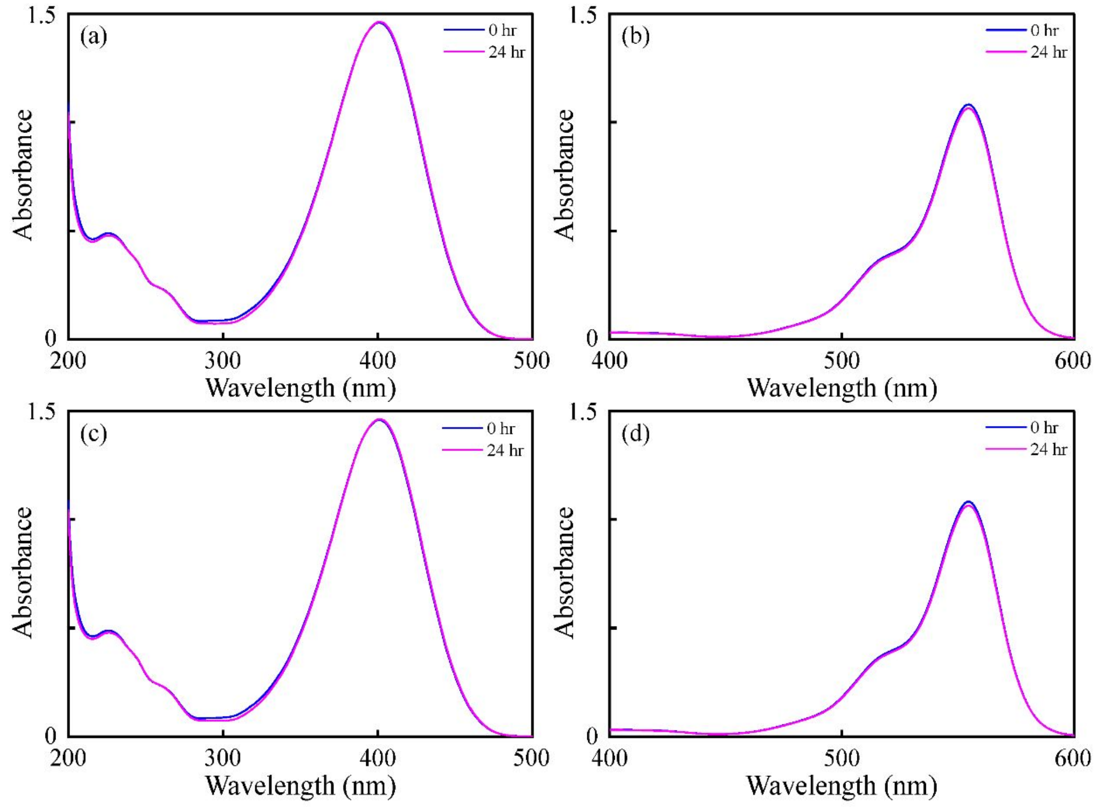

Figure S8. Catalytic degradation of (a) 4-NP and (b) RB by $NaBH_4$ in the absence of the TA-CuAu nanoparticles. Catalytic degradation of (c) 4-NP and (d) RB by the TA-CuAu nanoparticles in the absence of $NaBH_4$.

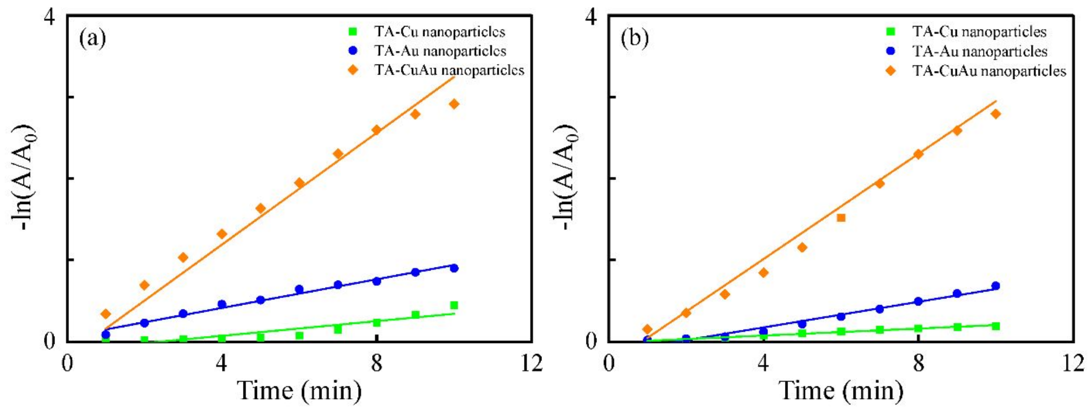

Figure S9. Plots of $-\ln(A/A_0)$ vs reaction time for the TA-Cu, TA-Au, and TA-CuAu nanoparticles with the absorbance of (a) 4-NP and (b) RB.

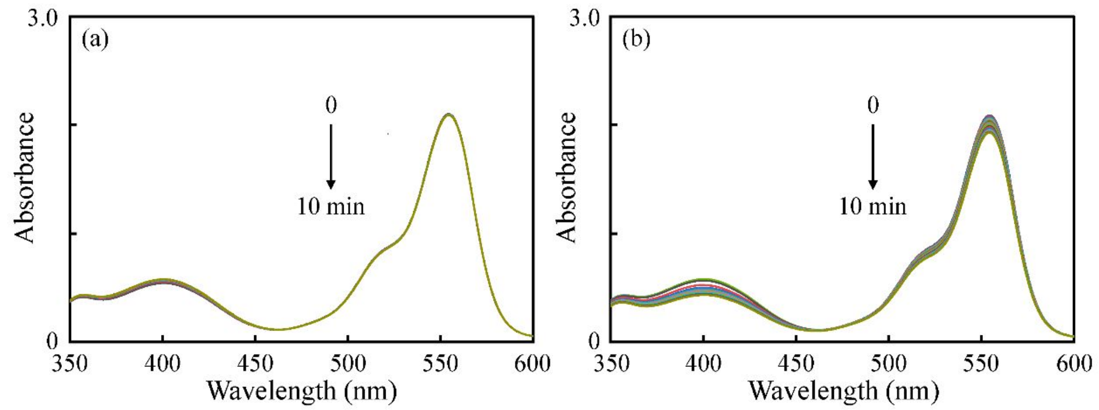

Figure S10. Simultaneous degradation of 4-NP (0.25 mM) and RB (0.1 mM) by the (a) TA-Cu and (b) TA-Au nanoparticles in the presence of $NaBH_4$.

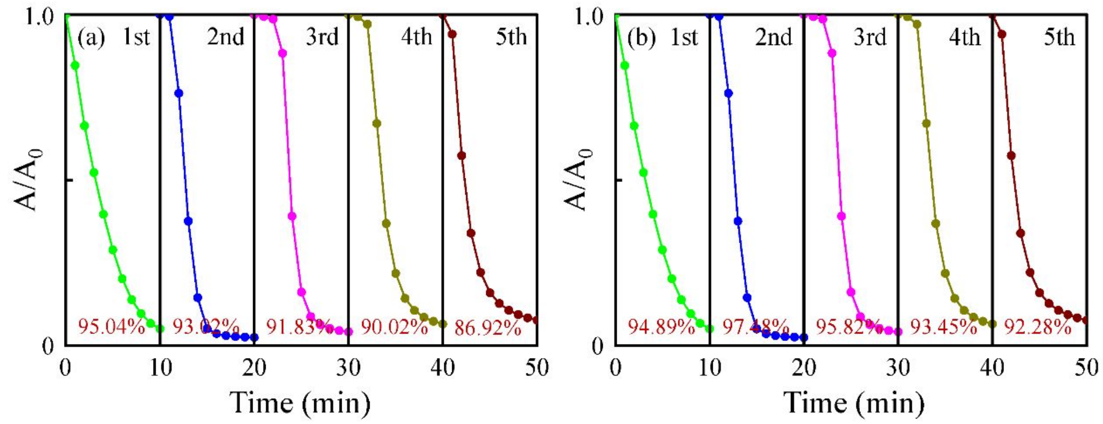

Figure S11. The reusability of the TA-CuAu nanoparticles as catalysts for five successive cycles of (a) 4-NP and (b) RB reduction.

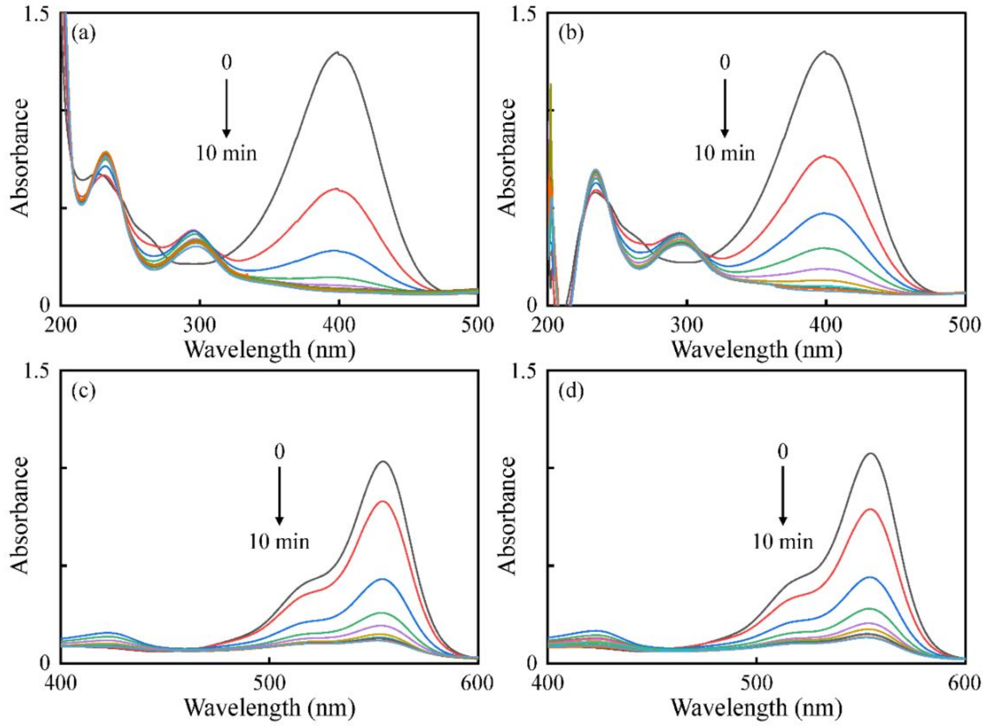

Figure S12. Time-dependent UV–Vis absorption spectra for the degradation of spiked (a, b) 4-NP and (c, d) RB by the TA-CuAu nanoparticles in the presence of $NaBH_4$ in (a, c) tap and (b, d) lake water samples.

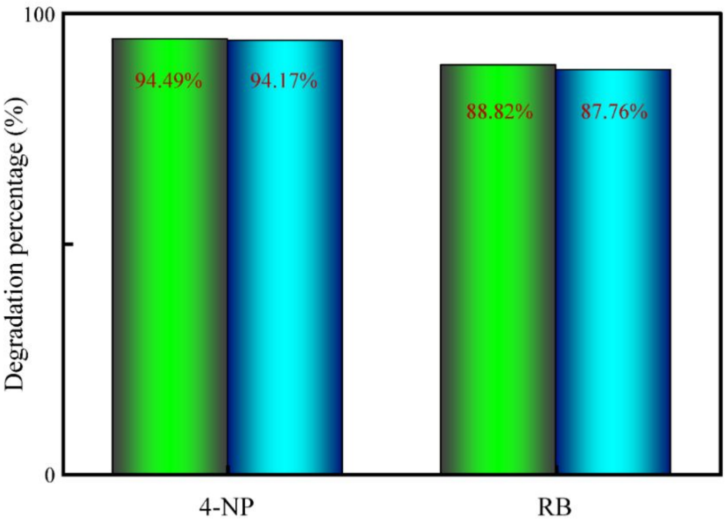

Figure S13. Degradation percentages of the TA-CuAu nanoparticles toward 4-NP and RB in the tap (green bar) and lake (blue bar) water samples.
